# 6.B. Round table: Insights from COVID-19: Preparing for and responding to the next health emergency

**DOI:** 10.1093/eurpub/ckac129.351

**Published:** 2022-10-25

**Authors:** Organised by: Institute for Health Metrics and Evaluation, Division of Country health Policies and Systems, WHO Europe, Chair persons: Maja Pasovic (USA), Elizabeth Serieux (USA)

## Abstract

The COVID-19 pandemic has stretched national health care systems to its limits, underscoring the importance of advance planning for health emergencies and the role of health data and evidence for informed policy interventions and actionable strategies. The experience with the pandemic has demonstrated the constraints of existing health information systems and the negative impacts that the lack of actionable and timely data can have on the implementation of rapid response measures during emergencies. This workshop aims to assist participants to become better prepared to deal with future health threats, such as pandemics, by exploring the role of trust in pandemic preparedness and response, effective communication strategies during a pandemic, and the use of qualitative research for evaluation of pandemic response. The specific objectives are to share and discuss: (a) results and conclusions derived from an online qualitative survey of experiences and lessons learned by member states across the WHO European Region; b) results and conclusions derived from an IHME study exploring the factors that contribute to / predict pandemic preparedness, and (c) lessons learned in the process of developing and disseminating scientifically valid, timely evidence across the globe, to assist policymaking during a pandemic. Together, these topics present and explore what countries and populations can do to effectively mitigate the negative effects of a pandemic or other health emergencies. These strategies and lessons learned include building trust, communicating clearly and succinctly, and using qualitative methods and data to evaluate pandemic response. Collectively, these are all geared at improving the capacity to address such threats and unexpected emergencies in the future. The panelists will also reflect on successful tools and strategies developed to aid policymakers and country governments in their response to the COVID-19 emergency. The workshop will be in the form of a 60-minute round table discussion with three panelists. Each panelist will make a brief presentation on their specific area of research, followed by a discussion between the panel members and audience.

**Key messages:**

• Trust, transparent communication, and reliable and timely data are critical aspects of both pandemic preparedness and response.

• The importance of qualitative evaluation for exploring what worked during the pandemic and what innovations should become part of an enhanced health information system in the future is essential.

**Speakers/Panellists:**

**Maja Pasovic**

Institute for Health Metrics and Evaluation, Seattle, USA

**Elizabeth Serieux**

Institute of Health Metrics and Evaluation, Seattle, USA

**David Novillo-Ortiz**

WHO Regional Office for Europe, Copenhagen, Denmark

